# Survivorship research in advanced gynecological cancer: A scoping review of cohort studies

**DOI:** 10.1002/cam4.6744

**Published:** 2023-11-27

**Authors:** Tracey DiSipio, Emma Pearse, Susan Jordan

**Affiliations:** ^1^ School of Public Health The University of Queensland Brisbane Queensland Australia

**Keywords:** advanced cancer, disparity, gynecological cancer, survivorship, systematic review

## Abstract

**Background:**

Recent calls to action highlight the need to address gaps in our understanding of survivorship for those living with advanced gynecological cancer to support optimal care. To ensure future research fills these knowledge gaps, we need to understand the breadth of existing survivorship research in this patient group, including the outcomes assessed, the populations included and the duration and retention in follow‐up.

**Methods:**

We conducted a systematic scoping review searching PubMed, PsychINFO, and CINAHL during the month of November 2022 to identify prospective cohort studies measuring survivorship outcomes among participants with advanced (stage III–IV) gynecological cancer, or in cohorts in which ≥50% of participants had advanced cancer, or which provide results separately for patients with advanced cancer. Articles were screened, and data extracted using a standard form.

**Results:**

We assessed 33 articles from 21 unique studies, which overall included 6023 participants with gynecological cancer. Of these, 45% had cervical cancer, 44% ovarian, 10% endometrial/uterine, and 1% vaginal/vulvar cancer. The most frequently measured survivorship outcome was quality of life. Of the 33 articles, most reported on participant age (*n* = 31), but relatively few reported on comorbidities (*n* = 10), physical status (*n* = 6), ethnic background (*n* = 4), the country of birth (*n* = 2), or the area of participant residence (*n* = 2). None included details on indigenous status. Recruitment proportions ranged from 48% to 100%. Retention proportions ranged from 15% to 97%.

**Conclusion:**

Our findings highlight gaps in survivorship research for advanced gynecological cancers and emphasize the need for future studies to include and describe the experiences of diverse and underrepresented groups.

## Graphical abstract

Recent calls to action highlight the need to address gaps in our understanding of survivorship for those living with advanced gynecological cancer to support optimal care. Findings highlight gaps in survivorship research for advanced gynecological cancers and emphasize the need for future studies to include and describe the experiences of diverse and underrepresented groups.

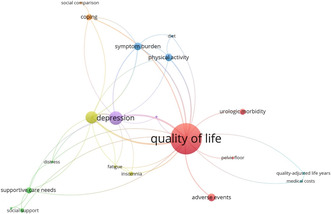

## 1 INTRODUCTION

Understanding how people diagnosed with advanced/metastatic gynecological cancer live with and manage the consequences of their disease and treatment side effects is important so that they can be optimally supported throughout the survivorship journey. The goals of care for these patients are a balance between treatment (i.e., to prolong life without expectation of cure) and palliation (i.e., to manage symptoms and optimize quality of life). Survivorship care, encompassing all aspects of health and wellbeing, should be considered early on and personalized to meet individual needs.^1^ However, little is known about long‐term experiences. In fact, the National Institutes of Health have identified as a priority longitudinal cancer survivorship research in general, and research into the long‐term needs of people living with advanced/metastatic cancer specifically.^2^, ^3^ These reports follow on from earlier calls for research to address gaps in our understanding of survivorship needs for those living with advanced/metastatic cancer.^4^, ^5^ Furthermore, an analysis of National Institutes of Health grants focused on patients living with advanced/metastatic cancers identified just one study focused on the survivorship needs of individuals living with advanced/metastatic *gynecological* cancer.^6^

There was an estimated 1.4 million females diagnosed with a gynecological cancer worldwide in 2020.^7^ Although information on stage is not universally available, statistics from the United States Surveillance, Epidemiology, and End Results (SEER) program estimate that for the period 2010–2019, between 28.8% (uterine) to 74.6% (ovarian) of gynecological cancers had spread (i.e., regional or metastatic disease).^8^ In the United States, survival rates for gynecological cancer decline considerably with increasing stage at diagnosis.^8^ To illustrate, for the period 2012–2018, uterine cancer had a five‐year relative survival of 81.3%, which decreases to 69.8% and 18.4% for regional and distant disease, respectively.^8^ Ovarian and vaginal cancers had the lowest five‐year relative survival rates of 50%, likely on account of the large proportions diagnosed with regional/distant disease; survival decreased to 30.8% and 25.9% for distant ovarian and distant vaginal disease, respectively.^8^ With an aging population, the number of cancer cases is expected to rise for most gynecological cancer types,^9^, ^10^ further highlighting the need to understand survivor experiences for optimal care planning.

Observational, prospective survivorship research is required to fill the evidence gaps. To do so in a way that produces meaningful and generalizable knowledge, it is important to ensure that longitudinal research considers design features that are inclusive of underrepresented groups and enhance recruitment and retention. This will offer maximum reach when translating evidence gathered to inform precision survivorship care. We use the term ‘underrepresented,’ which often includes older adults, indigenous, culturally and linguistically diverse (CALD), and regional/rural populations who are not proportionately represented in research.^11^ Researching advanced cancer is challenging because of the nature of the disease. Research on common barriers to enrolment and retention cite accessing patients (e.g., a clinical decision not to approach eligible patients, patients being discharged before being approached) and patient refusal (e.g., too unwell, severe distress, uncertainty regarding cancer stage) as common challenges.^12^, ^13^, ^14^, ^15^, ^16^ Additional barriers have been identified for recruiting underrepresented groups into cancer clinical trials, such as lack of trial awareness (e.g., lack of culturally appropriate information), lack of opportunity to participate (e.g., age, comorbidity, and functional status exclusion criteria), and the decision to refuse participation (e.g., mistrust of research).^11^ However, there is little information available on how these factors might influence participation of advanced cancer patients from underrepresented groups into observational longitudinal research.

The aim of this systematic scoping review (ScR) is to scope the body of literature available regarding survivorship among patients diagnosed with advanced gynecological cancer. The objectives of this ScR were to investigate (i) inclusion of advanced gynecological cancer patients in observational, prospective, cohort studies, (ii) survivorship outcomes reported (e.g., quality of life, lifestyle behaviors, health care service use), (iii) inclusion of underrepresented groups (i.e., age, comorbidity, indigenous, CALD, regional/rural status), and (iv) recruitment and retention proportions.

## 2 METHODS

We conducted and reported this review according to the Preferred Reporting Items for Systematic reviews and Meta‐Analyses extension for Scoping Reviews (PRISMA‐ScR) guidelines.^17^

### 2.1 Search strategy

A comprehensive search of relevant databases including PubMed, PsychINFO, and CINAHL was conducted in November 2022 by one investigator (TD) using a search strategy with a combination of terms relating to cohort studies, cancer, gynecological cancer, and advanced disease (see Supplementary Material S1). Search terms were adapted for use in each bibliographic database in combination with database specific filters (humans; female), with no restrictions on publication period or language. Additional articles were identified by hand searching reference lists of included articles.

### 2.2 Eligibility criteria and screening

Inclusion criteria were based on study design, participants, outcomes, and publication language. We included studies that were observational, prospective, cohort studies of people with advanced (stage III–IV) gynecological cancer. If studies included other stages, we included those in which at least 50% of patients were diagnosed with advanced cancer, or where results were provided separately for patients with advanced cancer. We used a broad definition of survivorship outcomes to encompass as many aspects of life after a cancer diagnosis and the survivor experience as possible including, but not limited to, quality of life, cancer and treatment symptom burden, other concerns (e.g., financial hardship), patterns and quality of care, lifestyle behaviors. Studies that only report on survival rates without including survivor experiences were not included. Papers published in English were included.

Covidence^18^ was used as the screening management tool. One investigator (TD) screened titles and abstracts for relevance against inclusion criteria. Full text articles were retrieved for titles/abstracts meeting the inclusion criteria, or where additional information was required to determine eligibility. Two investigators (TD and EP) independently reviewed the full text articles using the hierarchy of criteria for exclusion (see Supplementary Material S2). Disagreements were resolved by discussion between investigators.

### 2.3 Data extraction and synthesis of results

A data extraction form was created and piloted by two investigators (TD and EP) to ensure that key information to address the research objectives was captured. Relevant information that was extracted included study characteristics (i.e., first author, publication year, country the study was conducted in, study methodology, gynecological cancer site(s), sample size (where a study included mixed cancer sites, the number of gynecological cancers was abstracted), stage, time since diagnosis at baseline, year of recruitment, average study duration (including follow‐up of survivorship outcomes)), study outcomes (survivorship outcome, measurement instrument), participant characteristics (age, residential location, indigenous status, ethnicity, comorbidity, physical status), and recruitment and retention rates (including contextual data on exclusion criteria, reasons for declining to participate, reasons for attrition). One investigator (EP) independently extracted the data, and a senior investigator (TD) reviewed the data extraction for clarity and accuracy. Any queries about the data were resolved through discussion between the investigators. Methodological quality appraisal of the included papers was not performed as the latest guidance for conducting scoping reviews does not recommend performing an assessment of methodological limitations.^19^

#### 2.3.1 Study characteristics

Study characteristics were summarized as counts with percentages for categorical variables and means with distribution for continuous variables. Where a study included mixed cancer sites, characteristics specific to gynecological cancers were abstracted. Where the number (percent) of participants with advanced cancer was not available for the gynecological cancer of interest, the study average was applied. Where a range was provided for time since diagnosis at baseline, the average was reported. Study duration was noted for the length of follow‐up of survivorship outcomes.

#### 2.3.2 Survivorship outcomes

To identify survivorship topics, a keyword co‐occurrence network visualization analysis was conducted using author keywords (or Indexed/MeSH keywords if author keywords were not assigned). The investigators (TD and EP) cleaned the list of keywords in Endnote by removing terms referring to study methods and merging synonyms. Keywords were imported into VOSviewer, version 1.6.16^20^ for network visualization analysis. Each keyword is shown as a node and the size of the node indicates the frequency of that keyword (i.e., the larger the size of the node, the more frequent the keyword). Keywords that co‐occur frequently are clustered together and are located closer together in the network.

#### 2.3.3 Participant characteristics

Counts and percentages were used to summarize categorical characteristics and average age (distribution) was reported as per each publication.

Recruitment and retention: Recruitment proportions were calculated as the total number of participants recruited divided by the number of eligible participants. Retention proportions were calculated as the number of active participants at the last follow‐up for survivorship outcomes divided by the number of participants recruited at baseline. Individual study recruitment and retention at the end of follow‐up of survivorship outcomes are presented in forest plots; binomial proportion 95% confidence intervals (95% CI) for individual studies were calculated (Stata metaprop command).^21^ Due to the heterogeneity of included cancers, stage, and duration of follow‐up, proportions were not pooled. Where more than one article was published from a study, recruitment proportions were abstracted from the paper using the largest sample size, and retention proportions for the longest follow‐up of survivorship outcomes. Where a study included mixed cancer sites, only the gynecological cancers were included; where this information was not available, recruitment and retention proportions have been extrapolated using the overall study results. Where details were not available, protocol papers and previous publications were sourced; recruitment and retention proportions were not included if information was unavailable and therefore not included in the synthesis of results.

## 3 RESULTS

### 3.1 Screening and study selection

**FIGURE 1:**
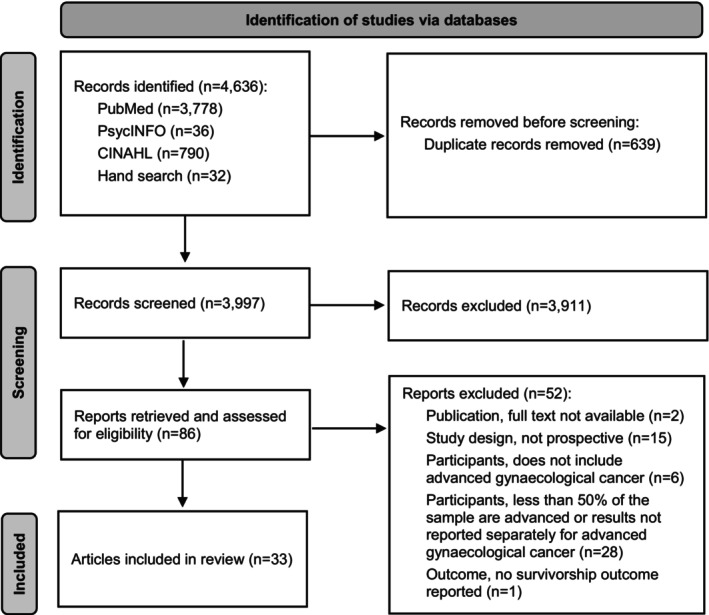
PRISMA flowchart.

Our database search identified 4636 potentially relevant articles. After removal of duplicates, 3997 titles/abstracts were screened (Figure 1). Of these, we excluded 3911 after title/abstract review. The remaining 86 full text records were retrieved and screened for eligibility. Of these we excluded 53 records for the following reasons: the full text was not available (*n* = 2); the study was not an observational, prospective cohort (*n* = 15); the study had no participants with advanced gynecological cancer (*n* = 6); in the study, less than 50% of the participants had advanced disease or results were not reported separately for advanced gynecological cancer (*n* = 29); or no survivorship outcome was reported (*n* = 1) in the study. In total, we included 33 articles and extracted data for synthesis.

### 3.2 Study characteristics

A summary of the characteristics of included studies are presented in Table 1 and characteristics of individual articles are displayed in Table 2.

**TABLE 1:** Summary characteristics of the 21 unique studies included^a^.

	*n* (%)	
Cancer site		
Cervical	4 (19)	
Ovarian	9 (43)	
Vulvar	1 (5)	
Various gynecological sites	5 (24)	
Various cancer sites	2 (9)	
Study methodology		
Quantitative	18 (86)	
Mixed method	3 (14)	
Location of study		
Asia	6 (29)	
Australia	4 (19)	
Europe	4 (19)	
North America	2 (9)	
South America	1 (6)	
United Kingdom	2 (9)	
International	2 (9)	
	**Mean**	**Minimum, maximum**
Sample size	293	(13, 1416)
Proportion of sample with advanced stage disease, %	46	(25, 100)
Time since diagnosis at baseline, months	9	(0,19)
Study duration, months	24	(3, 60)

^a^ Number of articles/unique study. Australian Ovarian Cancer Study (AOCS) = 5; An International Study on MRI‐Guided Brachytherapy in Locally Advanced Cervical Cancer (EMBRACE) = 3; Lymphoedema Evaluation in Gynecological cancer Study (LEGS) = 2; Ovarian cancer Prognosis and Lifestyle (OPAL) = 4; Sowerbutts = 2; Vaz = 2; Single paper/study = 15.

**TABLE 2:** Characteristics of included articles sorted alphabetically by cancer site and author (*N* = 33).

Author (year) Country; Study title	Sample size (*N*) and stage (%)	Recruitment year(s), time since diagnosis at baseline, and study duration	Participant characteristics (%)	Survivorship outcome(s)	Measurement instrument
*Cervical cancer*					
Lajer (2002)^22^ Denmark	*N* = 177 I: 15% II: 30% III: 54% IV: 1%	Recruitment: 1987–1991 Baseline: on admission for receiving radiotherapy Duration: 60 months	Median age: 59 years (range 22–86)	Late urologic morbidity (3+ months after radiotherapy)	Comparable to the Franco‐Italian glossary for grading complications due to radiation treatment
Lapitan (2011)^23^ Philippines	*N* = 205 IIIB–IVA: 100%	Recruitment: 2004–2009 Baseline: not available Duration: 12 months	Mean age: 48.2 years (range 26–71; SD 9.6) Comorbidity present: 23.5%	Quality of life	FACT‐G
Mahantshetty (2017)^24^ India; EMBRACE	*N* = 94 IIB: 33% IIIB: 58.5% IVA: 8.5%	Recruitment: 2009–2013 Baseline: during treatment Duration: 60 months (median 39 months)	Median age: 49 years (IQR 42–55)	Late toxicities (>90 days after end of treatment)	CTCAE (clinician‐assessed)
Mantegna (2013)^25^ Italy	*N* = 227 ECC: 46.3% LACC: 53.7%	Recruitment: 2007–2010 Baseline: within 1 week of diagnosis Duration: 24 months	Median age: 50.0 years (range 27.3–82.0) Comorbidity present: 23.8%	Anxiety and depression Quality of life	HADS EORTC QLQ‐C30 (GHS) and CX24
Pötter (2021)^26^ Europe, Asia, and North America; EMBRACE	*N* = 1341 IB1: 9.2% IB2: 8.9% IIA1: 2.8% IIA2: 2.3% IIB: 51.7% IIIA: 1.0% IIIB: 14.2% IVA: 2.5% IVB: 7.3% Missing: 0.1%	Recruitment: 2008–2015 Baseline: during treatment Duration: 60 months (median 51 months)	Median age: 49 years (IQR 41–60)	Late morbidity (3+ months after the end of treatment)	CTCAE (clinician‐assessed)
Spampinato (2021)^27^ International; EMBRACE	*N* = 1216 IB: 19.2% IIA: 5.4% IIB: 56.3% IIIA: 1.2% IIIB: 15.1% IVA: 2.8%	Recruitment: 2008–2015 Baseline: during treatment Duration: 48 months	Median age (no bladder wall infiltration): 49 years (IQR 21–91) Median age (bladder wall infiltration): 54 years (IQR 27–77) Comorbidity present: 28.5%	Urinary frequency and incontinence	CTCAE (clinician‐assessed) EORTC QLQ‐C30 and CX24 (patient)
*Ovarian cancer*					
Beesley (2011)^28^ Australia; AOCS‐QoL AOCS‐Lifestyle	N = 507 I–II: 32.2% III–IV: 65.8% Missing: 2.0%	Recruitment: 2005–2007 Baseline: 3 months to 5 years (median 26 months) Duration: 24 months	Mean age: 58 years (SD 10)	Physical activity Depression and anxiety Quality of life	HADS FACT‐O
Beesley (2013)^29^ Australia; AOCS‐QoL	*N* = 185 I–II: 24% III–IV: 76%	Recruitment: 2005–2007 Baseline: 6–12 months Duration: 24 months	Mean age: 59 years (SD 10) Residential location: Major city: 61% Inner regional: 26% Outer regional: 10% Remote/very remote: 3%	Supportive care needs	SCNS‐SF34
Beesley (2014)^30^ Australia; AOCS‐QoL PROSPECT	*N* = 172 I–II: 4% III–IV: 96%	PROSPECT: Recruitment: 2003–2005 Baseline: 1 month to 20 years (median 15 months) Duration: 24 months AOCS‐QoL: Recruitment: 2005–2007 Baseline: 3 months to 5 years (median 26 months) Duration: 24 months	Mean age at progression: 60 years (SD 10) Recurrent ovarian cancer	Quality of life	FACT‐O
Beesley (2018)^31^ Australia; OPAL	*N* = 634 I: 19% II: 9% III: 53% IV:12% Unknown: 7%	Recruitment: 2012–2015 Baseline: immediately after diagnosis Duration: 24 months	Median age: 61 years (range 21–79)	Coping strategies Anxiety and depression Quality of life	Brief‐COPE scale HADS FACT‐G and FACT‐O
Beesley (2020)^32^ Australia; OPAL	*N* = 527 I/II: 28% III/IV: 72%	Recruitment: 2012–2015 Baseline: during chemotherapy Duration: 24 months	Mean age: 60 years (SD 10.4) Comorbidity present: 25%	Anxiety and depression Sleep disturbance Fatigue Quality of life	HADS ISI FACIT‐F FACT‐G
Chan (2003)^33^ China	*N* = 17 IIIc: 24% IV: 76%	Recruitment: 1997–2000 Baseline: pre‐chemotherapy Duration: 24 months	Mean age: 54.6 years (SD 7.6) Median age: 57 years WHO performance status: 1: 35% 2: 65%	Quality of life (follow‐up to 12 months)	EORTC QLQ‐C30
Ding (2007)^34^ China	*N* = 75 I: 18.7% II: 20.0% III: 46.7% IV: 14.7%	Recruitment: 2001–2003 Baseline: during chemotherapy Duration: 9 months	Mean age: 49.55 years (range 17–70; SD 10.11)	Quality of life	FACT‐O
Gordon (2010)^35^ Australia AOCS	*N* = 85 I–II: 14% III: 74% IV: 11% Unknown: 1%	Recruitment: 2003–2005 Baseline: close to diagnosis Duration: 24 months	Mean age: 60.3 years (SD 9.9)	Quality of life Quality‐adjusted life years Medical costs	SF‐36 SF‐6D
Hansen (2020)^36^ Australia OPAL	*N* = 512 I: 27.5% II: 11.3% III: 53.9% IV: 7.2%	Recruitment: 2012–2015 Baseline: median = 29 days Duration: 48 months	Mean age: 59.1 years (SD 10.4) Comorbidity present: 23.4%	Healthy lifestyle Physical activity Diet Quality of life Symptom burden	Healthy Lifestyle Index Active Australia Survey FFQ FACT MOST
Inci (2021)^37^ Germany RISC‐GYN trial	*N* = 155 I–II: 17.6% III–IV: 82.4%	Recruitment: 2015–2017 Baseline: pre‐surgery Duration: 3 months	Age: <65 years: 64.5% ≥65 years: 35.5% ASA physical status grade >2: 31.2% ECOG performance status >1: 7.7% Comorbidity present >2: 29%	Health‐related quality of life (cross‐sectional analysis pre‐surgery)	EORTC QLQ‐C30
Jones (2021)^38^ Australia LEGS	*N* = 110 I: 23.6% II: 12.7% III: 48.2% IV: 12.7% Unknown: 2.7%	Recruitment: 2008–2011 Baseline: pre‐diagnostic surgery Duration: 24 months	Mean age: 60.72 years (SD 10.68; range 33–88) ECOG performance status: 0: 83.6% 1: 14.5% 2: 1.8% Birth country: Australia: 71.8% Other: 27.3% Unknown: 0.9%	Physical activity	Active Australia Survey
Morrell (2012)^39^ Australia	*N* = 13 III: 10% IV: 3%	Recruitment: not available Baseline: at diagnosis, first recurrence, subsequent recurrence, refractory, or progressive disease Duration: 30 months	Ethnic background: Anglo‐Australian: 53.8% United Kingdom: 23% Non‐English: 23%	Social comparison Anxiety and depression Quality of life	Interviews HADS FACT‐O
Price (2013)^40^ Australia AOCS‐QoL	*N* = 217 I–II: 7% III–IV: 93%	Recruitment: 2002–2006 Baseline: 3–55 months (mean 25.8 months) Duration: 24 months	Mean age: 62.6 years (SD 10.0) Residential location: Major city: 62% Regional/remote: 38%	Physical symptoms Coping styles Quality of life	FACT‐O items LORT and MAC FACT‐O
Ross (2020)^41^ Australia; OPAL	*N* = 894 I–II: 28% III–IV: 72%	Recruitment: 2012–2015 Baseline: 50 days Duration: 36 months	Mean age: 60 years (SD 11) Comorbidity present: 26%	Insomnia Quality of life	ISI FACT‐G
Sowerbutts (2020)^42^ England	*N* = 20 III–IV: 100%	Recruitment: 2016–2017 Baseline: in hospital, admitted with inoperable malignant bowel obstruction Duration: 15 months	Not available	Experiences of parenteral nutrition	Interviews
Sowerbutts (2019)^43^ England	*N* = 20 I: 5% II: 0% III: 80% IV: 15%	Recruitment: 2016–2017 Baseline: 27.5 months (range 1–184 months) Duration: 15 months	Mean age: 67 years (SD 7.5) ECOG performance status: 0: 5% 1: 25% 2: 35% 3: 15% 4: 0 Unknown: 20%	Patients' and family caregivers' experiences of home parenteral nutrition Nutritional status	Interviews Body mass index (self‐reported height and weight) and body composition (CT imaging)
Sundar (2022)^44^ Australia, India, United Kingdom; SOCQER‐2	*N* = 247 IIIA–IIIB: 9% IIIC: 56% IV: 35%	Recruitment: 2015–2016 Baseline: pre‐surgery Duration: 24 months	Age: ≤65 years: 57.9% >65 years: 42.1% ECOG performance status: 0: 43% 1: 46% 2+: 11% Comorbidity index: 0–2: 64% 3+: 36%	Quality of life	EORCT QLQ‐C30 and OV28
Zhou (2016)^45^ United States; ACS SCS‐I	*N* = 365 Localized: 28% Regional: 20% Distant: 52%	Recruitment: 2000–2005 Baseline: 1 year (average 1.3 years) Duration: 24 months	Mean age: 56.9 years Race/ethnicity: Non‐Hispanic White: 90% Other: 10%	Health‐related quality of life Physical symptoms	SF‐36 RSCL‐M
*Vulvar cancer*					
Jones (2016)^46^ United Kingdom	*N* = 23 IB: 65% II: 4% III: 22% IV: 9%	Recruitment: 2007–2009 Baseline: pre‐treatment Duration: 12 months	Mean age: 59.9 years (SD 15.3; range 23.8–86.6)	Health‐related quality of life Pelvic floor outcomes	EORCT QLQ‐C30 SF‐36 ePAQ‐PF
*Various cancer sites*					
The ACTION Study Group (2017)^47^ Southeast Asia; The ACTION study	Various cancer sites, *N* = 5249 Cervix: 598 Ovary: 123 Uterus: 127 Other: 4401 Stage among females, *N* = 3631 I: 9% II: 27% III: 19% IV: 7% None: 5% Missing: 32%	Recruitment: 2012–2013 Baseline: 12 weeks Duration: 12 months	Age (females): <45 years: 31% 45–54 years: 33% 55–64 years: 25% ≥65 years: 12% missing: <1% Comorbidity present (females): 24%	Health‐related quality of life Anxiety and depression	EORTC QLQ‐C30 EQ‐5D HADS
Fleming (2020)^48^ Australia; LEGS	Various gynecological sites, *N* = 408 Endometrial: 235 Ovarian: 114 Cervical: 37 Vulva/vaginal: 22 *N* = 408 I: 59% II: 10% III: 20% IV: 7% Missing: 4%	Recruitment: 2008–2011 Baseline: pre‐surgery Duration: 24 months	Mean age: 60 years (SD 11.4) Birth country: Australia: 65% Other: 26% Missing: 9%	Physical activity Quality of life	Active Australia Survey FACT‐G
Klapheke (2020)^49^ United States	Various gynecological sites, *N* = 248 Cervical: 11 Ovarian: 64 Uterine: 173 N = 248 Localized: 52% Regional/Distant: 44% Unknown: 4%	Recruitment: 1998–2014 Baseline: mean 12.54 (±7.11) months Duration: 24 months	Mean age: 74.1 years (SD 5.83) Race/ethnicity: White: 77.4% Asian or Pacific Islander: 9.3% Black or African American: 7.3% Hispanic: 5.2%	Functional impairment Health‐related quality of life	ADL SF‐36 VR‐12
Shafiq (2022)^50^ Singapore; COMPASS	Various cancer sites, *N* = 345 Gynecological: 22 Other: 323 *N* = 22 IV: 100%	Recruitment: 2016–2018 Baseline: not available Duration: until death	Age (all): <60 years: 46.1% ≥60 years: 53.9%	Physical symptom burden Psychological distress	FACIT‐Pal HADS
Tsao (2022)^51^ Taiwan	Various gynecological sites, *N* = 111 Cervical: 19 Endometrial: 24 Ovarian: 62 Uterine: 6 *N* = 111 I: 18% II: 18% III: 41.5% IV: 22.5%	Recruitment: 2019–2021 Baseline: pre‐treatment Duration: 6 months	Mean age: 52 years (SD 11; range 19–81) Comorbidity present: 42%	Supportive care needs Social support Hope	SCNS‐SF34 NSSQ‐T HHI
Vaz (2011a)^52^ Brazil	Various gynecological sites, *N* = 107 Cervical: 68 Endometrial: 39 *N* = 107 I–II: 36.5% III–IV: 63.5%	Recruitment: 2005–2009 Baseline: pre‐radiotherapy Duration: 36 months	Median age: 60 years (range 21–75)	Quality of life	WHOQOL‐BREF
Vaz (2011b)^53^ Brazil	Various gynecological sites, *N* = 107 Cervical: 68 Endometrial: 39 *N* = 107 I–II: 36.5% III–IV: 63.5%	Recruitment: 2005–2009 Baseline: pre‐radiotherapy Duration: 36 months	Median age: 60 years (range 21–75) Race/ethnicity: White: 46% Non‐white: 54%	Menopausal and sexual symptoms Quality of life	CTCAE (researcher‐assessed) WHOQOL‐BREF
Vogt (2021)^54^ Germany; APM‐Project	Various cancer sites, *N* = 500 Ovarian: 13 Other: 487 *N* = 13 Incurable: 100%	Recruitment: 2014–2016 Baseline: pre‐anticancer therapy Duration: 12 months	Mean age (all): 64.2 years (range 25–89) ECOG performance status (N = 500): 0: 20.6% 1: 50.0% 2: 21.0% 3: 6.0% 4: 0.6% Unknown: 1.8%	Distress Anxiety and depression Quality of life Symptom burden Importance and satisfaction with daily life Supportive care needs	Distress Thermometer PHQ‐4 FACT‐G FACT‐G items SEIQoL Modified SCNS‐25

*Note*: World Health Organization (WHO) performance status classification: 0, able to carry out all normal activity without restriction; 1, restricted in strenuous activity but ambulatory and able to carry out light work; 2, ambulatory and capable of all self‐care but unable to carry out any work activities, up and about more than 50% of waking hours; 3, symptomatic and in a chair or in bed for greater than 50% of the day but not bedridden; 4, completely disabled, cannot carry out any self‐care, totally confined to bed or chair [^55^].

American Society of Anaesthesiologists (ASA) physical status classification system: I, a normal healthy patient; II, a patient with mild systemic disease; III, a patient with severe systemic disease; IV, a patient with severe systemic disease that is a constant threat to life; V, a moribund patient who is not expected to survive without the operation; VI, a declared brain‐dead patient whose organs are being removed for donor purposes [^56^].

Eastern Cooperative Oncology Group (ECOG) performance status: 0, fully active and able to carry on all pre‐disease performance without restriction; 1, restricted in physically strenuous activity but ambulatory and able to carry out work of a light or sedentary nature; 2, ambulatory and capable of all self‐care but unable to carry out any work activities, up and about more than 50% of waking hours; 3, capable of only limited self‐care, confined to bed or chair more than 50% of waking hours; 4, completely disabled, cannot carry on any self‐care, totally confined to bed or chair; 5 dead [^55^].

Abbreviations: ACS SCS‐I, American Cancer Society Study of Cancer Survivors‐I; ACTION Study, ASEAN CosTs In Oncology (ASEAN, Association of Southeast Asian Nations); ADL, Activity of Daily Living; AMP‐Project, Arbeitsgemeinschaft Palliativmedizin; AOCS‐QoL, Australian Ovarian Cancer Study‐Quality of Life; ASA, American Society of Anaesthesiologists; Brief‐COPE Coping Orientation to Problems Experienced Inventory; COMPASS, Cost Of Medical care of Patients with Advanced Serious illness in Singapore; CTCAE, Common Terminology Criteria for Adverse Events; ECC, early cervical cancer; ECOG, Eastern Cooperative Oncology Group; EMBRACE, An International Study on MRI‐Guided Brachytherapy in Locally Advanced Cervical Cancer; EORTC QLQ‐C30, European Organization for Research and Treatment Quality of Life Questionnaire of Cancer Patients; EORTC QLQ‐CX24, European Organization for Research and Treatment Quality of Life Questionnaire Cervical Cancer; EORTC QLQ‐O28, European Organization for Research and Treatment Quality of Life Questionnaire Ovarian Cancer; ePAQ‐PF, electronic Personal Assessment Questionnaire‐Pelvic Floor; FACIT‐F, Functional Assessment of Chronic Illness Therapy‐Fatigue; FACIT‐Pal, Functional Assessment of Chronic Illness Therapy‐Palliative Care; FACT‐G, Functional Assessment of Cancer Therapy‐General; FACT‐O, Functional Assessment of Cancer Therapy‐Ovarian; FFQ, Food Frequency Questionnaire; HADS, Hospital Anxiety and Depression Scale; HHI, Herth Hope Index; IQR, interquartile range; ISI, Insomnia Severity Index; LACC, locally advanced cervical cancer; LEGS, The Lymphoedema Evaluation in Gynecological cancer Study; MAC, Mental Adjustment to Cancer; NSSQ‐T, Norbeck Social Support Questionnaire; OPAL, Ovarian cancer Prognosis and Lifestyle; PHQ‐4, Patients Health Questionnaire; RISC‐GYN Trial, Role of Predictive Markers for Severe Postoperative Complications in Gynecological Cancer Surgery; RSCL‐M, Rotterdam Symptom Checklist‐Modified; SCNS‐SF34, Supportive Care Needs Survey‐Short Form; SD, standard deviation; SEIQol, Schedule for the Evaluation of Individual Quality of Life; SF‐36, 36‐item Short Form Survey; SOCQER‐2 study, Surgery in Ovarian Cancer‐Quality of Life Evaluation Research; VR‐12, Veterans RAND 12‐item Health Survey; WHO, World Health Organization; WHOQOL‐BREF, World Health Organization's Quality of Life.

Of the 33 articles included, there were 21 unique studies with multiple articles reporting the results from the same study (Table 1). Across the 6023 participants with gynecological cancer included in the studies, 45% had cervical cancer, 44% ovarian, 10% endometrial/uterine, and 1% vaginal/vulvar cancer. On average, 46% of participants were diagnosed with advanced stage cancer; the proportions of advanced disease by cancer type were as follows: 60% ovarian, 36% cervical, 32% endometrial/uterine, and 22% vaginal/vulvar. Six studies included only women with advanced stage cancer: three ovarian, one cervical, and two included various cancer sites (gynecological cancers grouped together, and ovarian cancer in addition to other cancers).^23^, ^33^, ^39^, ^44^, ^50^, ^54^

Most studies (*n* = 18; 86%) were quantitative, and two‐thirds of the studies were conducted in either Asia, Australia, or Europe. The recruitment of study participants spanned from 1987 to 2021. The average number of enrolled participants across the studies was 287 (range 13 to 1341), and the average study duration was 24 months (range 3 to 60 months). Based on our inclusion criteria, the number of publications on advanced gynecological cancer survivorship issues has increased over time; two papers were published 20+ years ago, 10 in the following 10‐year period (2004 to 2013), and 21 papers in the past 10 years.

### 3.3 Survivorship outcomes

**FIGURE 2:**
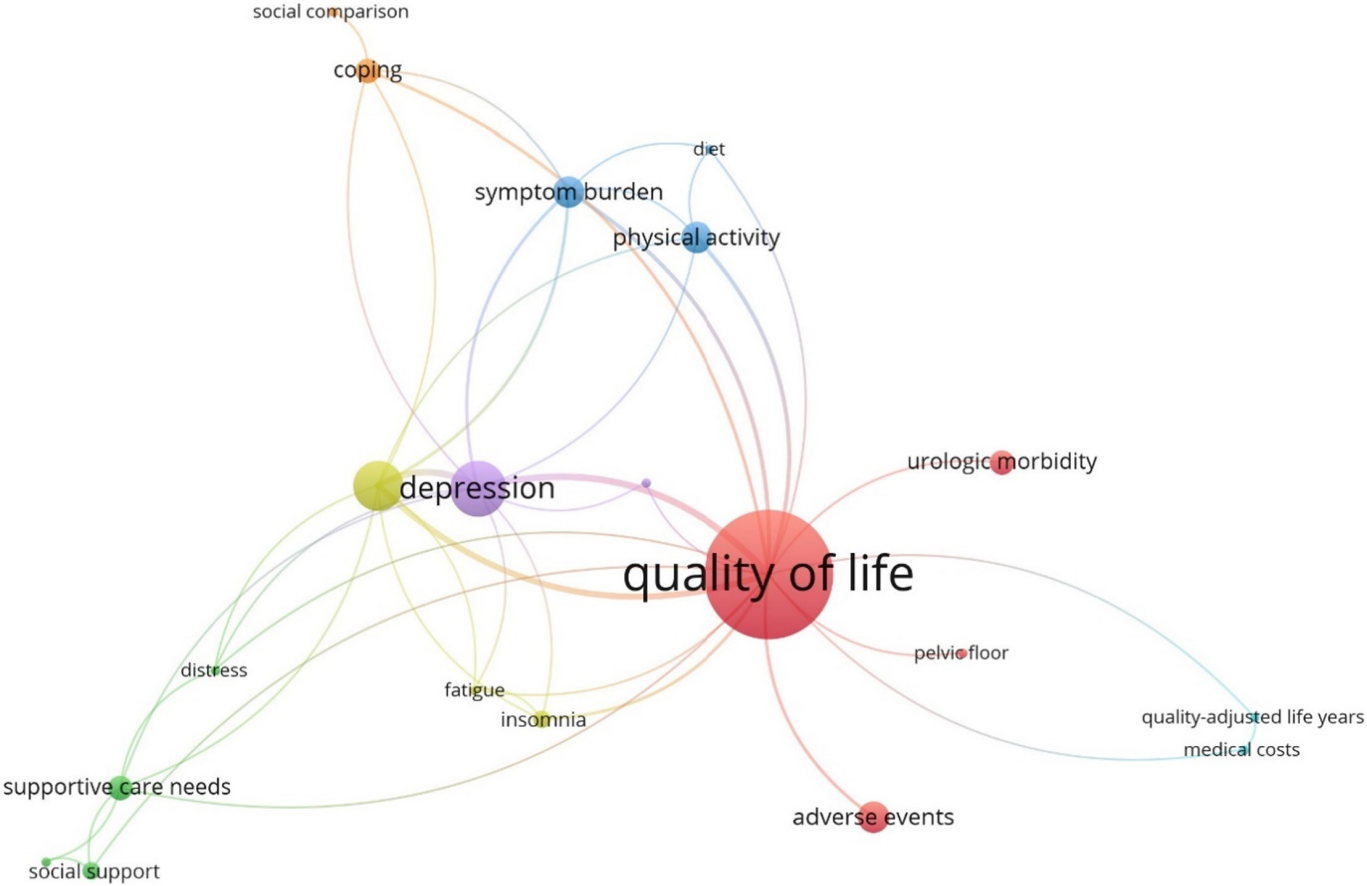
Keyword co‐occurrence network visualization.

The cohort studies addressed a selection of survivorship issues (Table 2). The most frequently occurring survivorship outcome was quality of life (*n* = 22 occurrences), as shown by the largest node in Figure 2. Keywords that frequently co‐occurred with quality of life were anxiety and depression. Cluster analysis identified seven keyword groups (represented in different colors in the figure). For example, topics related to morbidity were clustered in red with keywords quality of life, adverse events, pelvic floor, and urologic morbidity. Topics covering supportive care, social support, hope, and distress were clustered in green.

Survivorship outcomes were measured using standardized instruments. Quality of life was commonly measured by either the Functional Assessment of Chronic Illness Therapy (FACIT) or European Organization for Research and Treatment of Cancer (EORTC) suite of surveys. In addition to the general quality of life, 11 papers measured quality of life issues using cancer‐specific (e.g., cervical, ovarian), symptom‐specific (e.g., fatigue), or care‐specific (e.g., palliative care) modules.

### 3.4 Participant characteristics

Of the 33 articles included, 32 described at least one of the participant characteristics of interest including age (*n* = 31 articles), comorbidities (*n* = 10 articles), physical status (*n* = 6 articles), ethnic background (*n* = 4 articles), country of birth (*n* = 2 articles), and residential location (*n* = 2 articles). None of the included articles reported on indigenous status.

Based on the available information, the age of included participants ranged from 17 to 91 years. The proportion of participants with at least one comorbidity present ranged from 23% to 42%. In total, six articles reported on the physical status of participants according to the Eastern Cooperative Oncology Group/World Health Organization Performance Status (ECOG/WHO PS^55^); one article also provided status based on the American Society of Anesthesiologists (ASA) score.^56^ The participants in these studies reporting physical status all had ovarian cancer; three studies included only women with advanced stage disease; one article was based in a palliative care setting. The proportion of participants included who were fully active without restriction or restricted in strenuous activity only (ECOG/WHO PS = 0 or 1) ranged from 30% (for a study conducted in a palliative care setting) to 98%. The proportion of study participants from ethnic minority groups ranged from 10% (United States, non‐white) to 54% (Brazil, non‐white). Information on the country of birth of participants was provided in two papers reporting on one Australian study (LEGS) with 26% born overseas. Another two reports from a different Australian study (AOCS) described the residential location of participants, with 39% living outside major city areas.

### 3.5 Recruitment and retention

**FIGURE 3:**
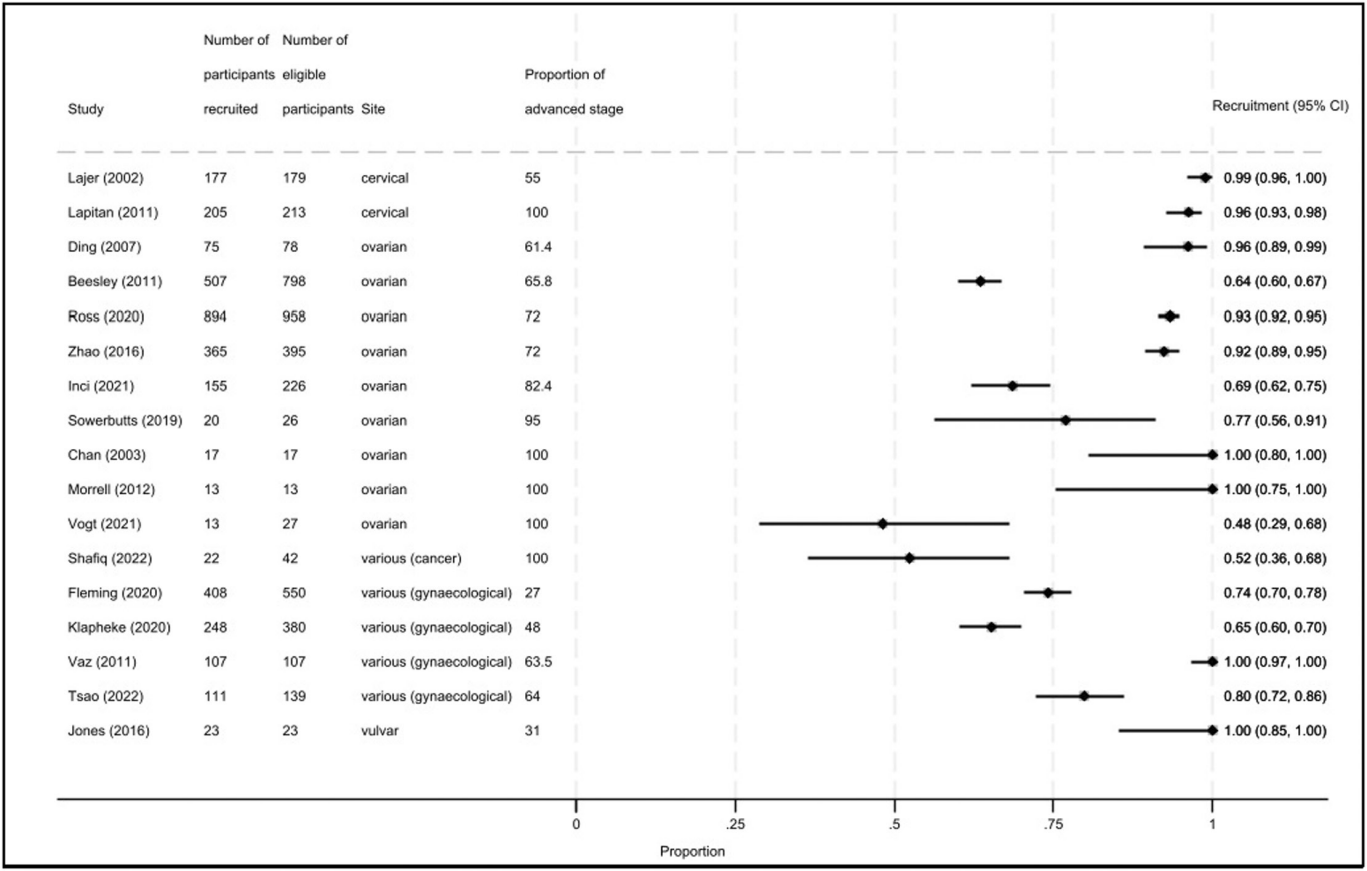
Forest plot for the recruitment proportions of included studies. Sorted alphabetically by cancer site and by proportion of advanced stage (lowest to highest). CI, confidence interval.

Recruitment proportions were available for 17 of the 21 studies (Figure 3). Recruitment proportions ranged from 48% (one study of cancer patients with incurable cancer, including ovarian cancer) to 100% (four studies). There was no clear pattern in recruitment proportions based on the proportion of the sample with advanced cancer. Three of the five studies that included only patients with advanced cancer achieved very high recruitment proportions (96%–100%). A common exclusion criterion cited was language (i.e., the ability to speak/read in the country's official language). Reasons for declining to participate were not well‐reported.

**FIGURE 4:**
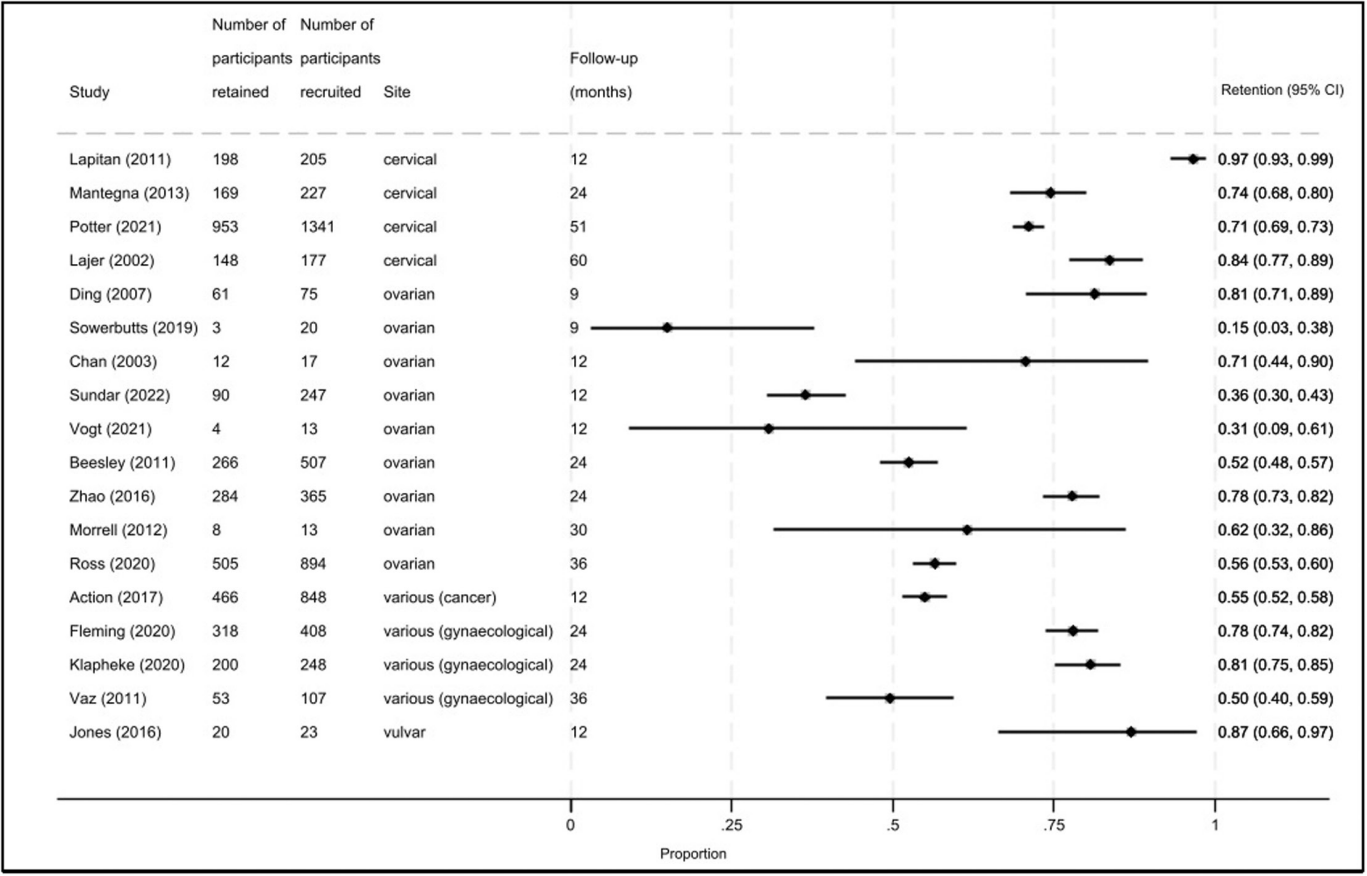
Forest plot for the retention proportions at the end of follow‐up for survivorship outcomes of included studies. Sorted alphabetically by cancer site and by duration of follow‐up (lowest to highest). CI, confidence interval.

Retention proportions were available for 18 of the 21 studies (Figure 4). Retention proportions ranged from 15% (in a study among ovarian cancer patients at 9 months follow‐up; 95% advanced stage) to 97% (in a study among advanced cervical cancer patients at 12 months follow‐up). There was no clear pattern in retention proportions by cancer site, proportion of the sample with advanced cancer, or follow‐up duration. Note that deaths were counted in retention proportions and death, or disease progression, were the most common reasons reported for attrition (listed in 18 papers). There was a lack of detail on other specific reasons for loss to follow‐up (listed in 10 papers) and withdrawal (listed in four papers).

## 4 DISCUSSION

The purpose of this scoping review was to comprehensively map the body of literature and identify existing gaps in survivorship research among patients with advanced gynecological cancer in prospective cohort studies. In total, 33 articles from 21 studies met the inclusion criteria with ovarian cancer the most frequently studied advanced gynecological cancer. We found there were a limited number of survivorship outcomes reported, although quality of life was commonly measured with comprehensive use of standardized measurement instruments. Our findings indicate that the inclusion of diverse and underrepresented groups was not comprehensively reported. Although recruitment and retention were generally well reported on, there was a lack of detail on the reasons patients declined to participate and the reasons for withdrawing. It is encouraging to observe an increase in the number of publications over time reporting on survivorship issues for patients diagnosed with gynecological cancers, yet gaps in our understanding remain. Therefore, we provide recommendations to improve the quality of reporting and embrace an inclusive understanding of this cohort's experience.

The identified studies included samples in which at least 50% of patients had advanced cancer or which reported results separately for participants with advanced stage disease. Only six studies focused exclusively on patients with advanced gynecological cancer: three specifically on ovarian cancer; one on cervical cancer, and two included various cancer sites (gynecological cancers grouped together, or ovarian cancer in addition to other cancers). The average proportion of participants with advanced gynecological cancer in the other 15 identified studies was 55%; however very few of the participants had advanced stage uterine, vaginal, or vulvar cancers. This may reflect the incidence of these cancers in that while uterine cancer is relatively common, it is less likely to be diagnosed at an advanced stage, while vaginal and vulvar cancers are rare.^8^ With information extremely limited, survivorship concerns among these patient groups needs to be explored. Of note, most of the research was conducted in high‐income countries and given that health systems and cultures vary by country, there is a need for survivorship research in low‐to‐middle income countries.

A broad range of cancer survivorship research topics have previously been mapped and will be relevant across cancer types.^57^ The unmet supportive care needs of people living with advanced cancer and their caregivers have also recently been reviewed.^58^ Whereas past reviews found survivorship topics covered included financial burden, disparity (e.g., race and ethnicity, rural, access to care), health care delivery, and communication, the present review found these survivorship topics were infrequently addressed in studies of patients with advanced gynecological cancer. Similar gaps in metastatic cancer survivorship research have been identified.^59^ Additional issues that may impact survivorship and therefore require attention includes nutritional status and physical condition; indeed, in the identified papers there was a lack of objective outcomes reported relating to these factors. The breadth of survivorship outcomes reported for cancer patients generally now need to be expanded to patients with advanced cancers specifically.

There are also several gaps in the current literature around *who* participated, with many patient characteristics not adequately reported. Most articles reported on the age of study participants and a broad age range was included. Based on information available on comorbidities and physical status (i.e., 30% or fewer articles), study participants were restricted to the ‘healthiest’ patients (i.e., 57%–77% of participants had no comorbidities and 30%–98% were fully active/restricted in strenuous activity only). Race/ethnicity or country of birth was reported in just six papers (five studies), residential location in two papers (one study), and none of the papers reported on indigenous status.

Standard reporting of participant characteristics to capture aspects of diversity would improve the quality of published research. Research reporting guidelines are common and one which would be relevant here is the CONSORT‐Equity extension which advocates for describing populations of interest across PROGRESS‐Plus characteristics (place of residence, race/ethnicity/culture/language, occupation, gender/sex, religion, education socioeconomic status, social capital; with additional characteristics as the “Plus”).^60^ Comprehensive reporting of participant sociodemographic and health characteristics is important to determine the generalizability of research findings, which are subsequently used to inform survivorship care and the design of precision survivorship care models.

People from CALD backgrounds often lack the opportunity to participate in research and, as such, the Australian Clinical Trials Alliance have released draft recommendations to increase engagement, involvement, and participation for people from CALD backgrounds in clinical trials.^61^ We extend this call to improve awareness and access to all study designs, and for including additional underrepresented groups. Policy changes will therefore be needed to support initiatives. Funding bodies (such as the National Institute for Health and Care Research and Wellcome in the United Kingdom, the National Institutes of Health in the United States) have issued guidance for funders and researchers to ensure people from underserved groups are represented in clinical research.^62^, ^63^, ^64^ Allocation of cancer funding also require reform. Disparities in cancer funding allocation have been reported in the United States, with gynecological cancers receiving significantly lower funding relative to their burden than other cancer sites,^65^, ^66^ as well as for cancers with high incidence rates among racial/ethnic minority populations.^67^ Efforts need to be made to ensure an equitable distribution of cancer research funding.

Conducting long‐term survivorship research among advanced cancer cohorts is challenging, however recruitment and retention proportions found here are encouraging. Recruitment proportions across 17 studies range from 48% to 100%. Retention proportions across 18 studies at the end of follow‐up for survivorship outcomes ranged from 15% to 97%. While recruitment and retention challenges have been documented,^12^, ^13^, ^14^, ^15^, ^16^ we are not aware of any review reporting results for observational research. Recruitment and retention among patients with advanced cancer participating in exercise interventions have been reported at 49% (range 15%–74%) and 76% (range 58%– 90%), respectively.^68^ Recruitment and retention were not correlated with the duration of exercise programs or frequency of exercise sessions. Recruitment and retention have also been reported for psychosocial interventions for patients and their caregivers at 33% (range 8%–100%) and 69% (range 16%–100%), respectively.^69^ Recruitment and retention to prospective cohort studies is challenging but possible. Efforts need to be made for involvement of diverse groups (see discussion above).

### 4.1 Strengths and limitations

Our scoping review has some potential limitations. This review excluded non‐English publications which may have subsequently excluded relevant studies published in other languages (thereby possibly overlooking research among diverse populations), study protocols (highlighting new studies being developed and implemented), articles that did not report stage of disease (potentially excluding relevant studies), and research among caregivers (who are included in the Institute of Medicine definition of survivor).^70^ We therefore acknowledge that it is possible that relevant studies were not included and that these experiences warrant separate review. To ensure our review was feasible, only one investigator performed the initial screening of titles/abstract of almost 4000 records; however, the investigator was purposively inclusive of records to avoid excluding potentially relevant records that may have been identified by a second reviewer.

### 4.2 Conclusions

The global burden of gynecological cancer is projected to increase both in terms of incidence (1.4 million to 1.95 million) and mortality (672,000 to 995,000) from 2020 to 2040.^71^ Yet high quality evidence (i.e., prospective cohort studies) tracking the risk factors for experiencing poor survivorship is lacking. The need for high quality cancer survivorship care was emphasized almost 20 years ago in the Institute of Medicine report, “From Cancer Patient to Cancer Survivor: lost in transition”^70^; while progress has been made, this scoping review highlights existing gaps in cancer survivorship research and provides recommendations to inform future research to address these needs.

## AUTHOR CONTRIBUTIONS

**Tracey DiSipio:** Conceptualization (lead); data curation (equal); formal analysis (equal); investigation (equal); methodology (equal); project administration (lead); supervision (lead); validation (equal); visualization (equal); writing – original draft (equal); writing – review and editing (equal). **Emma Pearse:** Data curation (equal); formal analysis (equal); investigation (equal); writing – review and editing (equal). **Susan Jordan:** Conceptualization (supporting); writing – review and editing (equal).

## FUNDING INFORMATION

Not applicable.

## CONFLICT OF INTEREST STATEMENT

None to declare.

## Supporting information


Data S1.

## Data Availability

The data underlying this article are available in the article and in its online Supplementary Material.
