# Supplementary material for: Survivorship research in advanced gynecological cancer: A scoping review of cohort studies
Source: Cancer Med. 2023 Nov 27;12(24):21779–97. doi: 10.1002/cam4.6744 (PMC10757120; doi:10.1002/cam4.6744)
Supplement: Supplementary file 1 — Data S1. [file CAM4-12-21779-s001.docx]

**SUPPLEMENTARY MATERIALS**

**Supplementary Methods**

S1. Search strategy

| **Cohort studies** | **Cancer** | **Gynecological** | **Advanced** |
| --- | --- | --- | --- |
| PubMed | | | |
| [Title/Abstract]  Cohort  Longitudinal  NOT [Title]  “Systematic review”  “Meta-analysis”  Metaanalysis  “Clinical trial”  “Controlled trial” | [Title/Abstract]  Cancer  Carcinoma  Malignan*  Neoplasm  Oncology  Tumour  Tumor | [Title/Abstract]  Gynecological  Gynaecological  Ovar*  Uter*  Cervi*  Endometrial  Vulva*  Vagin*  “Fallopian tube”  Placenta*  Genital | [Title/Abstract]  Advanced  Distant  Metasta*  “Late stage”  Recurren* |
| PsycINFO | | | |
| [Title or Abstract]  Cohort  Longitudinal  NOT [Title]  “Systematic review”  “Meta-analysis”  Metaanalysis  “Clinical trial”  “Controlled trial” | [Title or Abstract]  Cancer  Carcinoma  Malignan*  Neoplasm  Oncology  Tumour  Tumor | [Title or Abstract]  Gynecological  Gynaecological  Ovar*  Uter*  Cervi*  Endometrial  Vulva*  Vagin*  “Fallopian tube”  Placenta*  Genital | [Title or Abstract]  Advanced  Distant  Metasta*  “Late stage”  Recurren* |
| CINAHL | | | |
| [Title or Abstract]  Cohort  Longitudinal  AND NOT  “Systematic review”  “Meta-analysis”  Metaanalysis  “Clinical trial”  “Controlled trial” | [Title or Abstract]  Cancer  Carcinoma  Malignan*  Neoplasm  Oncology  Tumour  Tumor | [Title or Abstract]  Gynecological  Gynaecological  Ovar*  Uter*  Cervi*  Endometrial  Vulva*  Vagin*  “Fallopian tube”  Placenta*  Genital | [Title orAbstract]  Advanced  Distant  Metasta*  “Late stage”  Recurren* |

**Supplementary Methods**

S2. Hierarchy of exclusion

| 1 | Publication | Not published in English; could not source a translated copy. |
| --- | --- | --- |
| 2 | Publication | Full text not available; conference paper. |
| 3 | Study design | Not an observational, prospective, cohort study; requires repeated measures over time. Exclude protocols, commentaries, reviews, meta-analyses, other study designs. |
| 4 | Participants | Does not include females diagnosed with advanced gynecological cancer. |
| 5 | Participants | Less than 50% of the sample are advanced gynecological cancer or results are not reported separately for advanced gynecological cancer. |
| 6 | Outcome | No survivorship outcomes reported. |
